# Tissue Transglutaminase Knock-Out Preadipocytes and Beige Cells of Epididymal Fat Origin Possess Decreased Mitochondrial Functions Required for Thermogenesis

**DOI:** 10.3390/ijms23095175

**Published:** 2022-05-05

**Authors:** Kinga Lénárt, Csaba Bankó, Gyula Ujlaki, Szilárd Póliska, Gréta Kis, Éva Csősz, Miklós Antal, Zsolt Bacso, Péter Bai, László Fésüs, András Mádi

**Affiliations:** 1 Department of Biochemistry and Molecular Biology, Faculty of Medicine, University of Debrecen, Egyetem ter 1., H-4032 Debrecen, Hungary; kinga.lenart@med.unideb.hu (K.L.); szilard.poliska@med.unideb.hu (S.P.); eva.csosz@med.unideb.hu (É.C.); laszlo.fesus@med.unideb.hu (L.F.); 2 Doctoral School of Molecular Cell and Immune Biology, University of Debrecen, Egyetem ter 1., H-4032 Debrecen, Hungary; banko.csaba@med.unideb.hu; 3 Department of Biophysics and Cell Biology, Faculty of Medicine, University of Debrecen, Egyetem ter 1., H-4032 Debrecen, Hungary; zsolt.bacso@med.unideb.hu; 4 NKFIH-DE Lendület Laboratory of Cellular Metabolism, Department of Medical Chemistry, Faculty of Medicine, University of Debrecen, Egyetem ter 1., H-4032 Debrecen, Hungary; gyula.ujlaki@med.unideb.hu (G.U.); baip@med.unideb.hu (P.B.); 5 Department of Anatomy, Histology Embryology, Faculty of Medicine, University of Debrecen, Egyetem ter 1., H-4032 Debrecen, Hungary; greta@anat.med.unideb.hu (G.K.); miklos.antal@anat.med.unideb.hu (M.A.); 6 Research Center for Molecular Medicine, Faculty of Medicine, University of Debrecen, Egyetem ter 1., H-4032 Debrecen, Hungary

**Keywords:** browning, uncoupling protein-1, beige adipocytes, DIO3, SLC25A45

## Abstract

Beige adipocytes with thermogenic function are activated during cold exposure in white adipose tissue through the process of browning. These cells, similar to brown adipocytes, dissipate stored chemical energy in the form of heat with the help of uncoupling protein 1 (UCP1). Recently, we have shown that tissue transglutaminase (TG2) knock-out mice have decreased cold tolerance in parallel with lower utilization of their epididymal adipose tissue and reduced browning. To learn more about the thermogenic function of this fat depot, we isolated preadipocytes from the epididymal adipose tissue of wild-type and TG2 knock-out mice and differentiated them in the beige direction. Although differentiation of TG2 knock-out preadipocytes is phenotypically similar to the wild-type cells, the mitochondria of the knock-out beige cells have multiple impairments including an altered electron transport system generating lower electrochemical potential difference, reduced oxygen consumption, lower UCP1 protein content, and a higher portion of fragmented mitochondria. Most of these differences are present in preadipocytes as well, and the differentiation process cannot overcome the functional disadvantages completely. TG2 knock-out beige adipocytes produce more iodothyronine deiodinase 3 (DIO3) which may inactivate thyroid hormones required for the establishment of optimal mitochondrial function. The TG2 knock-out preadipocytes and beige cells are both hypometabolic as compared with the wild-type controls which may also be explained by the lower expression of solute carrier proteins SLC25A45, SLC25A47, and SLC25A42 which transport acylcarnitine, Co-A, and amino acids into the mitochondrial matrix. As a consequence, the mitochondria in TG2 knock-out beige adipocytes probably cannot reach the energy-producing threshold required for normal thermogenic functions, which may contribute to the decreased cold tolerance of TG2 knock-out mice.

## 1. Introduction

Tissue transglutaminase (TG2, EC 2.3.2.13) is a ubiquitous multifunctional protein [1,2]. It cross-links proteins covalently in a Ca^2+^-dependent manner through transamidase activity and forms isopeptide bonds between glutamine and lysine residues [3]. Moreover, TG2 can incorporate primary amines into glutamine residues of proteins, and it is also able to cleave the cross-links by isopeptidase activity [4]. In the absence of Ca^2+^, it binds GTP and takes closed conformation, and can act as a GTPase [5]. TG2 may also function as a G-protein (Ghα) transmitting a signal from 7-transmembrane receptors such as the α1B-adrenoceptor (α1B-AR) [6,7]. Functions of TG2 have been associated with numerous biological processes including regulation of the cytoskeleton, cell adhesion [8], and cell death [9] acting as catalytically active or just as an interacting protein partner [1,2,10]. It has been linked to several diseases [11], such as cancer [12,13], neurodegenerative disorders [14,15], fibrosis [16], and coeliac disease [17]. To unfold the complex biological function of TG2, knock-out mouse models (TG2^−/−^) have been made [18,19]. These animals are viable, and grow up to normal size and weight with no apparent abnormalities in organ functions [2]. Careful examinations have pointed to some abnormalities [20,21], particularly under stress and pathological conditions [2], revealing impaired wound healing [22], autoimmunity [23], and hyperglycemia [24].

It is well known that cold exposure or direct adrenergic stimuli can provoke the appearance of heat-producing beige cells in WAT through the process of browning [25]. Similar to brown adipocytes, the thermogenic function of beige cells relies on the activity of the mitochondrial uncoupling protein 1 (UCP1) that dissipates stored chemical energy in the form of heat by facilitating proton leak across the inner mitochondrial membrane and dissociating substrate oxidation from ATP production [26,27]. We showed previously that TG2^−/−^ animals had decreased cold tolerance as compared with TG2^+/+^ mice due to low mobilization of fatty acids and reduced browning mechanism in the epididymal white adipose tissue (EWAT) [28]. After cold exposure, TG2^−/−^ mice possessed a markedly whiter and higher amount of EWAT with larger sizes of adipocytes expressing a lower level of UCP1 protein as compared with TG2^+/+^ animals. Meanwhile, other investigated fat depots were utilized equivalently in the two strains during the cold exposure [28]. Thus, we wanted to learn more about the metabolism and the potential thermogenic function of TG2^−/−^ beige cells differentiated from preadipocytes of EWAT. Previously, it has been shown that TG2 plays a role in the differentiation processes of several cell types [1]. TG2 can regulate white adipocyte differentiation [29], and additionally, upregulation of TG2 has been shown in human BAT as compared with WAT [30]. We found that TG2^−/−^ preadipocytes could differentiate in the beige direction; however, the thermogenic capacity of generated TG2^−/−^ cells was significantly lower as compared with TG2^+/+^ beige cells due to mitochondrial dysfunctions which were apparent in preadipocytes as well, and could not be compensated by the differentiation process completely.

## 2. Results

### 2.1. Differentiation of TG2^−/−^ Preadipocytes Results in Altered Beige Adipocytes Phenotypically Similar to TG2^+/+^ Cells

**Figure 1:**
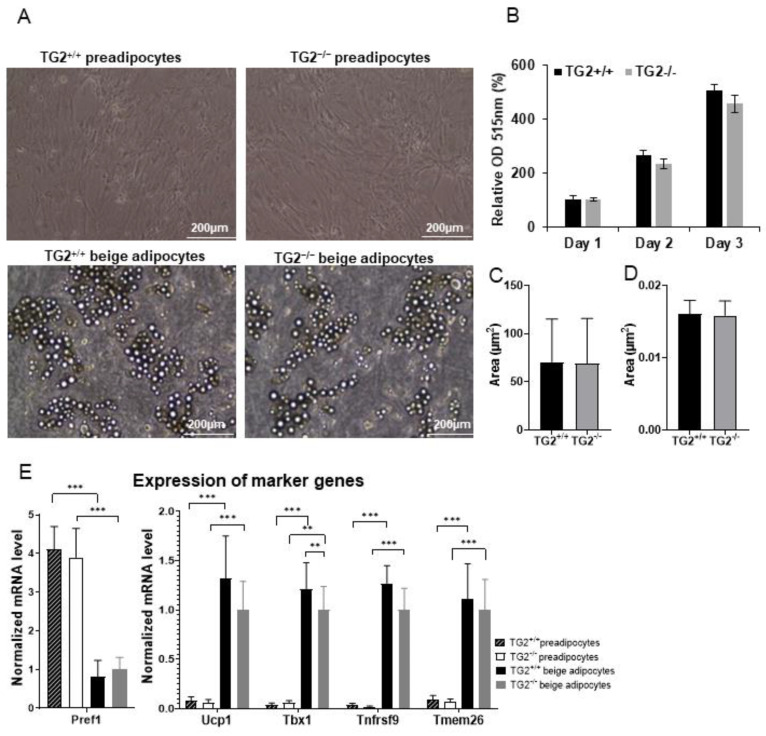
Differentiation of beige cells from TG2^+/+^ and TG2^−/−^ preadipocytes isolated from the epidydimal fat depots: (**A**) Typical microscopic view of confluent preadipocytes and differentiated beige cells, images were taken using EVOS FL Cell Imaging System, scale bars represent 200 μm; (**B**) proliferation capacity of TG2^+/+^ and TG2^−/−^ preadipocytes, cell density values were determined using Sulforhodamine B assays (*n* = 3); (**C**) average size of lipid droplets in beige cells; (**D**) total lipid content in beige cells; (**E**) gene expression value of the preadipocyte marker (*Pref1*) and beige marker genes (*Ucp1*, *Tbx1*, *Tnfrsf9*, and *Tmem26*) in preadipocytes and beige cells (*n* = 3). Columns represent the mean values ± SD. Statistical analyses were carried out using GraphPad Prism 7.0 version, by two-way ANOVA (Tukey’s multiple comparison test and Student’s *t*-test. ** *p* < 0.01, *** *p* < 0.001.

We isolated stromal vascular fractions (SVFs) from the EWAT of the mice. When the preadipocytes reached about 90% confluency, we started to differentiate them to the beige cell direction [31]. In 14 days, the cells accumulated multilocular lipid droplets and formed characteristics of in vitro differentiated beige adipocytes (Figure 1A). SRB assays showed that TG2^−/−^ preadipocytes proliferate at the same rate as TG2^+/+^ cells (Figure 1B), and software analyses of microscopic images revealed that the size of individual lipid droplets and the total fat content in TG2^−/−^ beige adipocytes were the same as in TG2^+/+^ cells (Figure 1C,D). We assessed the rate of the effectiveness of the differentiation process by measuring the expression of marker genes [32,33] in preadipocytes and beige cells. The expression of the preadipocyte marker Pref1 significantly decreased, while the beige markers *Ucp1*, *Tbx1*, *Tnfrsf9*, and *Tmem26* significantly increased in both TG2^+/+^ and TG2^−/−^ cells after completing the differentiation process, but the expression level of Tbx1 was lower in TG2^−/−^ beige adipocytes as compared with the TG2^+/+^ cells (Figure 1E).

### 2.2. Lack of TG2 Alters the Level of Mitochondrial Membrane Proteins and Energy Production

**Figure 2:**
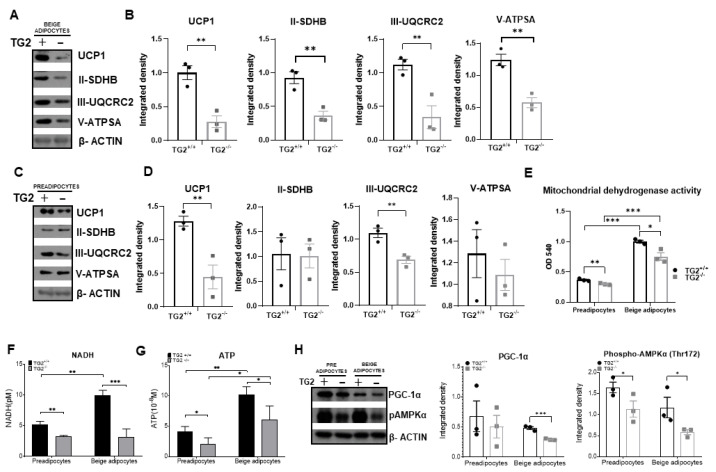
Detection of UCP1 and mitochondrial complex proteins and the levels of NADH and ATP in TG2^+/+^ and TG2^−/−^ cells together with PGC1α and pAMPK expressions: (**A**) Representative Western blots of UCP1, II-SDHB, III-UQCRC2, V-ATPSA of beige cells (*n* = 3); (**B**) quantitative analyses of Western blots of UCP1, II-SDHB, III-UQCRC2, and V-ATPSA proteins (*n* = 3); (**C**) representative Western blots of UCP1, II-SDHB, III-UQCRC2, and V-ATPSA of preadipocytes (*n* = 3); (**D**) quantitative analyses of Western blots of UCP1, II-SDHB, III-UQCRC2, and V-ATPSA proteins of preadipocytes; (**E**) mitochondrial dehydrogenases activity levels in preadipocytes and differentiated beige cells (MTT assays, *n* = 3); (**F**) NADH and (**G**) ATP content of preadipocytes and beige cells; (**H**) representative Western blots and quantitative analyses of phospho-AMPKα and PGC-1α in preadipocytes and beige cells. β-ACTIN was used as a loading control. Columns represent the mean values ± SD. Statistical analyses were performed using Student’s *t*-test. *, **, and *** indicate statistically significant differences at *p* < 0.05, *p* < 0.01, or *p* < 0.001, respectively. *n* = 3.

An increase in mitochondrial oxidative capacity plays an important role in the browning process of WAT, therefore, we examined the expression of inner mitochondrial membrane proteins responsible for ATP and heat production. Interestingly, we found that the expression of UCP1 at the protein level was significantly lower in TG2^−/−^ beige adipocytes as compared withTG2^+/+^ cells, and the levels of succinate dehydrogenase iron-sulfur subunit of complex II (II-SDHB), ubiquinol-cytochrome c reductase core protein 2 of complex III (III-UQCRC2), and the alpha subunit of ATP synthase in complex V (V-ATPSA) were also lower in these cells (Figure 2A,B). These results raised the possibility that some of the expression differences between TG2^+/+^ and TG2^−/−^ beige cells also exist in the preadipocytes from which they start to differentiate. Indeed, we detected significantly lower expressions of UCP1 and III-UQCRC in TG2^−/−^ preadipocytes, while no significant differences were detected in the cases of II-SDHB and V-ATPSA (Figure 2C,D). Moreover, mitochondrial dehydrogenase activity (Figure 2E), and the levels of NADH (Figure 2F), and ATP (Figure 2G) were all significantly lower both in the TG2^−/−^ preadipocytes and beige cells as compared with the corresponding TG2^+/+^ cells.

The co-activator PGC1α is the master regulator of mitochondrial networks and energy homeostasis in adipocytes [34], therefore, we investigated its expression and found that it was lower in the TG2^−/−^ beige cells (Figure 2H). It is known that there is a close association between the activation of AMPK by phosphorylation and the induction of PGC1α in adipocytes [35]. Accordingly, we observed reduced phospho-AMPK in TG2^−/−^ beige cells as well as in preadipocytes as compared with the TG2^+/+^ cells (Figure 2H).

### 2.3. TG2^−/−^ Preadipocytes and Beige Cells Generate Significantly Lower Membrane Potential in Mitochondria as Compared with TG2^+/+^ Cells

**Figure 3:**
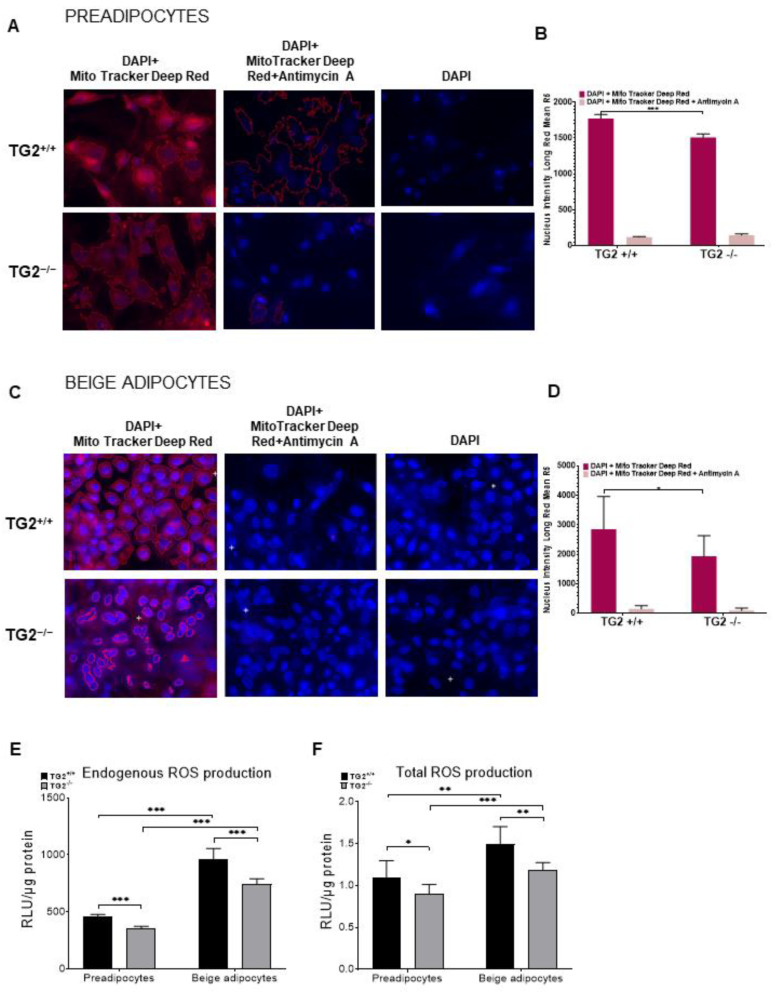
Mitochondrial membrane potential levels and ROS production in TG2^+/+^ and TG2^−/−^ cells: (**A**) Representative laser scanning cytometry images of TG2^+/+^ and TG2^−/−^ preadipocytes; (**B**) quantitative analyses of mitochondrial membrane potential in preadipocytes; (**C**) representative laser scanning cytometry images of TG2^+/+^ and TG2^−/−^ beige cells; (**D**) quantitative analysis of mitochondrial membrane potential in beige cells, DAPI staining was used to determine the number of cell nuclei, cells were stained with Mito Tracker Deep Red either in the absence or the presence of 10 μM antimycin; (**E**) endogenous and (**F**) total ROS production of preadipocytes and beige cells. Columns represent the mean values ± SD. Statistical analyses were performed using Student’s *t*-test. *n* = 4. * *p* < 0.05, ** *p* < 0.01, *** *p* < 0.001).

We carried out laser scanning cytometry (LSC) measurements using MitoTracker Deep Red to learn how changes in the inner mitochondrial membrane proteins affect the formation of the mitochondrial membrane potential in the investigated cell types. Mitochondrial retention of MitoTracker Deep Red depends upon mitochondrial membrane potential [36], and can accordingly be higher in cells with increased OXPHOS. To demonstrate that only intact cells take up MitoTracker dye, we added the electron transport inhibitor antimycin A as a control. We found that MitoTracker Deep Red fluorescence intensity was significantly lower in the TG2^−/−^ preadipocytes (Figure 3A,B) and beige adipocytes (Figure 3C,D) as compared with the TG2^+/+^ cells.

When the mitochondrial electron transport chain (ETC) generates the electrochemical proton gradient which drives ATP synthesis, it also induces the production of reactive oxygen species (ROS). The major sources of mitochondrial ROS production are the ubiquinone sites in complexes I and III [37] in which we detected expression differences (Figure 2B,D). Thus, we measured both the endogenous ROS production without any activation (Figure 3E) and the total ROS production capacity of the cells that they can generate after induction with PMA (Figure 3F). We detected lower ROS levels in TG2^−/−^ preadipocytes and beige cells as compared with the TG2^+/+^ cell types, indicating decreased activity of TG2^−/−^ mitochondria.

### 2.4. TG2^−/−^ Preadipocytes and Beige Cells Are Metabolically Hypometabolic as Compared with TG2^+/+^

**Figure 4:**
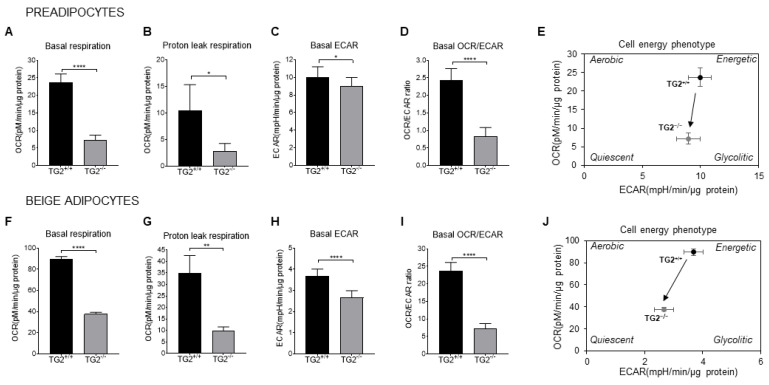
The bioenergetic profiles of TG2^+/+^ and TG2^−/−^ preadipocytes and beige cells: (**A**) Mitochondrial basal oxygen consumption rate (OCR); (**B**) proton leak OCR detected after addition of oligomycin; (**C**) basal extracellular acidification rate (ECAR); (**D**) OCR/ECAR ratio under basal conditions; (**E**) the energy phenotype profile (EPP) of preadipocytes; (**F**) mitochondrial basal OCR; (**G**) proton leak OCR detected after addition of oligomycin; (**H**) basal extracellular acidification rate (ECAR); (**I**) OCR/ECAR ratio under basal conditions; (**J**) the energy phenotype profile (EPP) of beige cells. OCR and ECAR were simultaneously measured using a Seahorse Bioscience XF-96 analyzer. Columns represent the mean values ± SD. Statistical analyses were performed using Student’s *t*-test. *n* = 4, * *p* < 0.05, ** *p* < 0.01, **** *p* < 0.0001.

Oxygen consumption rate (OCR) is an indicator of the ETC activity, therefore, we measured the mitochondrial OCR to test the functional capacity of preadipocytes and differentiated beige cells. We observed significantly lower basal respiration and proton leak respiration, which was related to heat production in the TG2^−/−^ preadipocytes (Figure 4A,B) and beige adipocytes (Figure 4F,G) as compared with the TG2^+/+^ cells. In parallel, we detected a significantly lower extracellular acidification rate (ECAR) both in TG2^−/−^ preadipocytes (Figure 4C) and beige adipocytes (Figure 4H) as compared with the TG2^+/+^ controls. In addition, the OCR/ECAR ratio at the basal conditions was also lower in the TG2 deficient cells as compared with the control preadipocytes (Figure 4D) and beige adipocytes (Figure 4I), indicating that TG2^−/−^ cells preferred oxidative phosphorylation less than glycolysis to produce energy. Analyses of the cell energy phenotypes revealed that TG2^+/+^ cells were characterized by a pronounced glycolytic and oxidative metabolism, as indicated by high ECAR and OCR as compared with the the TG2^−/−^ cells. Therefore, our results strongly suggest that cells lacking TG2 are hypometabolic relative to the wild-type controls (Figure 4E,J).

Next, we performed transmission electron microscopy (TEM) on preadipocytes to examine whether there were differences in the structure and the total mass of mitochondria (Supplementary Figure S1A). We found that the number of mitochondria and the total area of mitochondria in the cytoplasm of cells was the same in the TG2^+/+^ and TG2^−/−^ genotypes (Supplementary Figure S1B,C). We grouped the mitochondria according to their size, but we did not detect differences in any of the groups using this approach (Supplementary Figure S1D). Unfortunately, due to their triacylglycerol content, we could not examine the beige cells using TEM for technical reasons. We also investigated the level of the mitochondrial protein TOM20 and found that its expression was the same in TG2^+/+^ and TG2^−/−^ cells (Supplementary Figure S1E), further suggesting that there were no differences in the number and size of mitochondria between the two genotypes.

### 2.5. TG2^−/−^ Beige Adipocytes Have More Fragmented Mitochondria as Compared with TG2^+/+^

**Figure 5:**
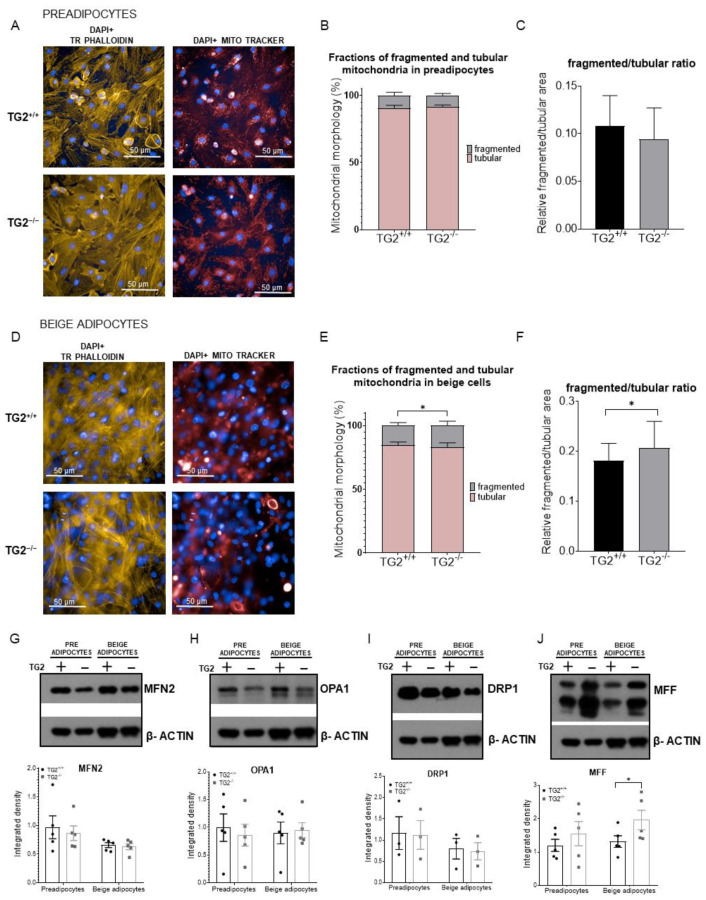
High content screening of the preadipocytes and beige cells, and detection of mitochondrial fission- and fusion-related proteins: (**A**) Representative high content screening images showing mitochondrial morphology in TG2^+/+^ and TG2^−/−^ preadipocytes, scale bars represent 50 μm; (**B**) fractions of fragmented and tubular mitochondrial morphology in preadipocytes (%); (**C**) quantitative analysis of mitochondrial morphology in preadipocytes; (**D**) representative high content screening images showing mitochondrial morphology in TG2^+/+^ and TG2^−/−^ beige cells; (**E**) fractions of fragmented and tubular mitochondrial morphology in beige cells (%); (**F**) quantitative analysis of mitochondrial morphology in beige cells, DAPI staining was used to determine the number of nuclei, the mitochondria were stained with Mito Tracker Deep Red either in the absence or presence of 10 μM antimycin A, Texas Red-X phalloidin was used to stain actin filaments for the detection of cell shapes (*n* = 3); (**G**–**J**) representative Western blot analyses and quantitative analyses of the mitochondrial fusion proteins (MFN2, OPA1) and mitochondrial fission proteins (DRP1, MFF) in preadipocytes and differentiated beige cells. β-ACTIN was used as a loading control. Columns represent the mean values ± SD. Statistical analyses were performed using Student’s *t*-test. *n* = 5. * *p* < 0.05.

Mitochondria continuously fuse and divide through the processes known as fusion and fission forming dynamic networks. To study the morphology of mitochondria in TG2^−/−^ cells in more detail, we performed high-content-screening analyses (HCS, Figure 5A,D), and classified the Mitotracker Deep Red-stained mitochondria as either tubular or fragmented using the software’s embedded pixel classification module. The fragmented and tubular mitochondria were segmented as fragmented and tubular classes on the MitoTracker Red channel images. Tubular mitochondrial morphology was dominant both in preadipocytes and beige cells (Figure 5B,E). Although the ratio of fragmented/tubular mitochondria was not different in preadipocytes (Figure 5C), we found significantly more fragmented mitochondria in TG2^−/−^ beige adipocytes as compared with the TG2^+/+^ beige cells (Figure 5F).

We further investigated this phenomenon at the protein level by measuring the expression of mitochondrial shaping proteins [38]. The expressions of fusion proteins MFN2 and OPA1, and the fission protein DRP1 were the same in both preadipocytes and beige cells (Figure 5G–I). However, in parallel with the HCS results, the expression of the mitochondrial fission factor MFF was significantly increased in the TG2^−/−^ beige adipocytes as compared with the TG2^+/+^ cells (Figure 5J).

### 2.6. TG2^−/−^ Beige Adipocytes Can Attenuate the Effect of Free Triiodothyronine

**Figure 6:**
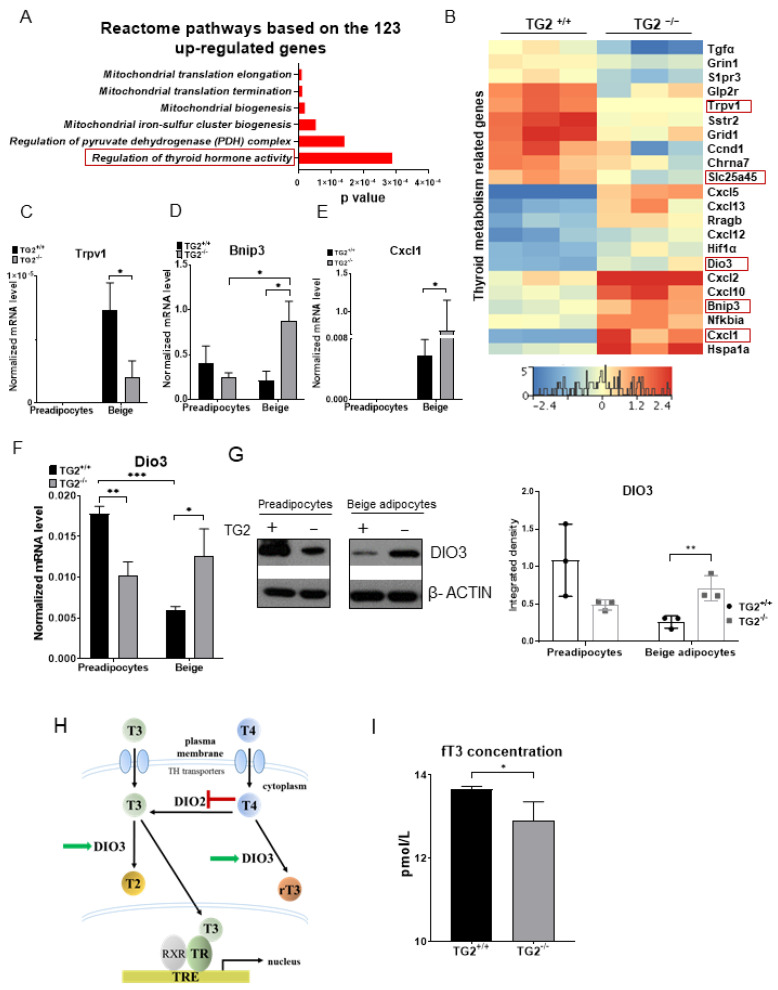
Upregulated mitochondrion-related genes in TG2^−/−^ beige cells as compared with TG2^+/+^: (**A**) Upregulated reactome pathways in the TG2^−/−^ beige samples, based on the gene ontology (GO, Reactome pathway) analysis; (**B**) heatmap of the thyroid metabolism-related genes in beige adipocytes, framed genes were selected for validation; (**C**–**F**) validation of the selected thyroid metabolism-related genes using RT-qPCR analyses; (**G**) representative Western blot analysis and quantitative analyses of DIO3 in preadipocytes and differentiated beige cells, β-ACTIN was used as loading control; (**H**) schematic representation of DIO3 in thyroid hormone inactivation, the panel is a modification of a figure from [40]; (**I**) fT3 concentration in the differentiation medium of the beige cells. Columns represent the mean values ± SD. Statistical analyses were performed using Student’s *t*-test. *n* = 3. * *p* < 0.05, ** *p* < 0.01, *** *p* < 0.001.

**Figure 7:**
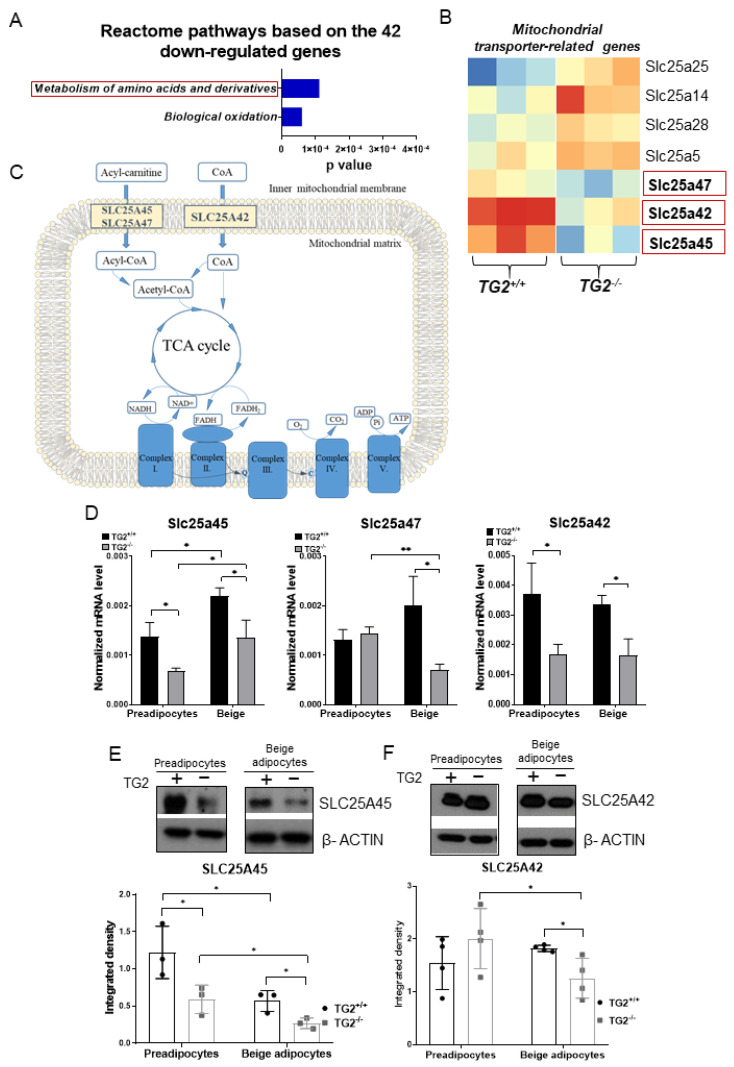
Downregulated mitochondrion-related genes in TG2^−/−^ beige cells as compared with TG2^+/+^: (**A**) Downregulated Reactome pathways in the TG2^−/−^ beige samples based on the gene ontology (GO, Reactome pathway) analysis; (**B**) heatmap of differentially expressed mitochondrial transporter genes in beige adipocytes, the framed genes were selected for validation; (**C**) schematic illustration of the roles of selected SLC transporters, the panel is a modification of a figure from [47]; (**D**) validation of the selected mitochondrial transporter genes using RT-qPCR analyses; (**E**) representative Western blot of SLC25A45 and quantitative analysis in preadipocytes and differentiated beige cells; (**F**) representative Western blot of SLC25A42 and quantitative analysis in preadipocytes and differentiated beige cells. Columns represent the mean values ± SD. Statistical analyses were performed using Student’s *t*-test. *n* = 3. * *p* < 0.05, ** *p* < 0.01.

To learn more about the molecular background of the observed differences between the phenotypes of TG2^+/+^ and TG2^−/−^ cells, we performed RNA-seq measurements to identify differentially expressed genes related to mitochondrial functions and browning. Our analyses revealed a total of 165 differences in beige adipocytes, including 123 upregulated and 42 downregulated genes in TG2^−/−^ beige cells. Concerning the upregulated genes in the TG2^−/−^ beige samples, gene ontology (GO, Reactome pathway) analysis showed that these genes were significantly enriched for mitochondrial biogenesis and especially the function of the thyroid hormone (Figure 6A). Thyroid hormone may also suppress some genes, consequently, among both the upregulated and downregulated genes, we identified those which could be regulated by the thyroid receptor (Figure 6B). From the genes downregulated in the TG2^−/−^ beige cells, we selected *Trpv1* (Figure 6C) and *Slc25a45* (Figure 7D) for validation using RT-qPCR. Among the genes upregulated in the TG2^−/−^ beige samples, we validated the expression of *Bnip3*, *Cxcl1*, and *Dio3* (Figure 6D–F). Importantly, we found that DIO3 protein expression correlated with the RT-qPCR result and it was higher in the TG2^−/−^ beige adipocytes (Figure 6G). DIO3 catalyzes the inactivation of the thyroid hormone by inner ring deiodination both of the prohormone thyroxine (T4) forming inactive reverse triiodothyronine (rT3) and the bioactive hormone T3 producing inactive T2 (Figure 6H) [39]. As the biologically effective T3 is a standard component of the differentiation cocktail and required for beige differentiation, we measured the levels of free T3 (fT3) in the supernatants of the beige adipocytes. We found that the fT3 concentration was significantly lower in the media of TG2^−/−^ beige cells as compared with the TG2^+/+^ controls (Figure 6I), reflecting the effect of higher DIO3 levels in the TG2^−/−^ cells.

### 2.7. Expression of Transporters of Acylcarnitine, CoA, and Certain Amino Acids into the Mitochondria Is Lower in TG2^−/−^ Cells as Compared with the TG2^+/+^ Controls

The gene ontology analysis showed that the downregulated genes in the TG2^−/−^ beige samples were significantly enriched for the metabolism and transport of amino acids and derivatives (Figure 7A). Some upregulated solute carriers (*Slc*s) genes could perform the function of the downregulated genes, therefore, we identified all seven differentially expressed *Slc*s (Figure 7B) and selected the three downregulated genes for validation using RT-qPCR (Figure 7C,D). SLC25A45 transports acylcarnitine, ATP, and amino acids across the inner membrane into the matrix [41,42,43], and its lower gene expression was successfully validated in both TG2^−/−^ preadipocytes and beige cells. SLC25A47 is a paralog of SLC25A45 and its gene expression level was lower in TG2^−/−^ beige cells but not different in preadipocytes. The third investigated carrier SLC25A42 can transport CoA and ADP into the mitochondria suggesting that it is also important for energy production [44,45,46]. We found that the *Slc25a42* gene expression was significantly lower in both TG2^−/−^ preadipocytes and beige cells as compared with the TG2^+/+^ control cells. Importantly, we found that the expression of SLC25A45 was lower in both TG2^−/−^ preadipocytes and beige cells also at the protein level (Figure 7E), while the expression of SLC25A42 protein was lower only in the TG2^−/−^ beige cells (Figure 7F).

As amino acid uptake is known to be required for the efficient thermogenic response of human mitochondria [48] and both SLC25A45 and SLC25A47 may also transport amino acids, we investigated the uptake and release of amino acids by the preadipocytes and beige cells. We detected remarkable production/consumption differences by comparing the contents of the fresh medium with the levels of the supernatants of cells for 48 h. On the one hand, TG2^+/+^ preadipocytes consumed significantly more His, Asn, Gln, Asp, Cys, Tyr, Met, and Phe as compared with the TG2^−/−^ cells (Supplementary Figure S2A). On the other hand, TG2^+/+^ beige cells consumed more Gln, Arg, and Met and released more Asn, Gly, Thr, Ala, and Trp than the TG2^−/−^ beige cells. Interestingly, TG2^−/−^ beige cells took up more Ser and produced more Pro as compared with the TG2^+/+^ controls (Supplementary Figure S2B). The most obvious difference was in the case of Gln, from which TG2^+/+^ cells took up much more than the TG2^−/−^ cells. Gln is one of the most important carbon sources for brown adipocytes [49], and it may have a similar function in beige cells as well. Gln can be easily converted to Glu, and then to the TCA intermediate alpha-ketoglutarate (AKG), resulting in NADH, and finally heat production.

## 3. Discussion

Mouse strains resistant to weight gain through increased brown and beige fat activity have demonstrated that activation of thermogenesis can be a promising approach to improve metabolic health and prevent weight gain [50,51]. Recently, we identified TG2 as a regulator in the regulation of the body temperature during cold exposure in an EWAT-specific manner. We demonstrated that brown adipose tissue (BAT) and subcutaneous white adipose tissue (SWAT) functions could support TG2^−/−^ animals during cold exposure, but decreased utilization and browning of EWAT was accompanied by decreased cold tolerance of the TG2^−/−^ mice as compared with TG2^+/+^ littermates [22]. Therefore, we assumed that TG2 had a possible role in the formation of beige cells with thermogenic functions as well. To substantiate the potential role of TG2 in the differentiation and operation of beige cells, we isolated and differentiated preadipocytes from EWAT of TG2^−/−^ animals and TG2^+/+^ littermates, and carried out comparative studies.

Beige precursor cells have to go through proliferation steps before robust browning can take place [52], and we found that the proliferation capacity of TG2^+/+^ and TG2^−/−^ preadipocytes were the same. The differentiated beige cells contained the same amount of lipids with similar droplet sizes. Moreover, there were no differences in the expression profiles of beige marker genes between the two genotypes, except the *Tbx1*, which was lower in the TG2^−/−^ differentiated cells. Transcription factor TBX-1 is known to control beige adipose tissue development and plays an important role in maintaining body temperature during cold exposure [53]. These results revealed that the TG2^+/+^ and TG2^−/−^ preadipocytes were able to differentiate in the beige direction using the protocol described in the literature [31].

Mitochondria play a key role in the metabolic processes including the tricarboxylic acid (TCA) cycle, oxidative degradation of fatty acids (β-oxidation), and formation of ATP by ETCs and V-ATPSA. Interestingly, we found lower expressions of ETC components and V-ATPSA in the TG2^−/−^ beige adipocytes. Mitochondria are also crucial in the generation of heat via the inner membrane protein UCP1 which uncouples respiration from ATP synthesis, and therefore, provokes energy dissipation in the form of heat while also stimulating high levels of fatty acid oxidation [27,28]. The notable observation that a preadipocyte expresses a terminal beige differentiation marker such as UCP1 is already known from the literature [54]. The protein expression of UCP1 was lower in TG2^−/−^ beige cells, suggesting that they have significantly lower heat-producing capacity as compared with TG2^+/+^. Indeed, the heat-producing proton leak respiration was significantly reduced in TG2^−/−^ cells which may be caused by their lower UCP1 expression.

We detected lower expression of III-UQCRC2 and decreased NADH dehydrogenase activity in TG2^−/−^ precursors as compared with TG2^+/+^ precursors, suggesting that the functions of mitochondrial ETC and ATP synthesis are defective at several sites in TG2^−/−^ preadipocytes and, consequently, in differentiated beige cells. Reduced energy production capacity was confirmed by detecting significantly lower NADH and ATP levels in both TG2^−/−^ preadipocytes and beige cells. We also demonstrated that the lack of TG2 led to lower mitochondrial membrane potential in preadipocytes and beige cells. Interestingly, while the altered structure and function of ETC could be expected to increase the ROS levels [46,47], we found the opposite, i.e., a significant decrease in ROS production in the TG2^−/−^ cells suggesting lower mitochondrial metabolism. It has been shown in other cell types that TG2 is implicated in maintaining the homeostasis of ETC and energy production. Deletion of TG2 in mice caused significant deregulation of the respiratory complexes I and II and a reduction of ATP production in mouse embryonic fibroblasts (MEFs) [55,56,57,58]; it was shown that PDI activity of TG2 is important for the formation of disulfide bridges in the ATP synthase complex and other key components of the ETC. However, our results on lower NADH and ROS levels suggest that reduced mitochondrial membrane potential and ATP production are also due to low levels of available fuels in TG2^−/−^ mitochondria. Indeed, analysis of the cell energy phenotypes revealed that TG2^+/+^ preadipocytes and beige cells were characterized by pronounced oxidative and glycolytic metabolism indicated by high OCR levels and ECAR as compared with the corresponding TG2^−/−^ cells and, therefore, were defined as the “energetic” cells. In contrast, our results strongly suggest that preadipocytes and beige cells are hypometabolic in the lack of TG2.

The metabolism of mitochondria depends on the balance of fusion and fission processes. Fusion leads to the formation of tubular, while fission yields fragmented structures of mitochondria. Our HCS results showed that the tubular mitochondrial morphology is the most characteristic of both preadipocytes and beige cells, however, there are significantly higher fractions of fragmented mitochondria in the TG2^−/−^ beige cells as compared with the TG2^+/+^ controls. We were able to demonstrate an important molecular factor behind this phenomenon, as we detected a significantly higher expression of MFF in TG2^−/−^ beige cells. MFF is an outer mitochondrial membrane protein that forms a complex with the GTPase DRP1 and promotes mitochondrial fragmentation [59]. Mitochondrial fragmentation is known to associate with depolarization, significantly decreased capacity of respiration, and reduced ATP production [60,61]. Interestingly, altered mitochondrial morphology and functionality have also been shown in TG2^−/−^ MEFs containing more fragmented and depolarized mitochondria [55]. We also found that the expression of the PGC1α, a driver protein of mitochondrial biogenesis, was significantly lower in the TG2^−/−^ beige cells. In line with this result, we also observed significantly lower expression of active phospho-AMPK in TG2^−/−^ cells. AMPK is a crucial sensor of the energy status of the cell, becoming phosphorylated and activated when the AMP/ATP ratio is high and triggering a wide range of catabolic pathways directed to increase cellular levels of ATP [62]. Activated AMPK directly phosphorylates and activates PGC1α [63,64,65]. Our data indicate that several processes that contribute to mitochondrial energy production and thermogenesis are impaired by the lack of TG2.

To learn more about the possible explanations of the obtained results, we carried out RNA-seq experiments and compared gene expression profiles of TG2^+/+^ and TG2^−/−^ beige cells. A similar investigation was performed in MEFs demonstrating that most of the differentially expressed genes were enriched in clusters related to cytoskeleton, actin regulation, and extracellular matrix regulation [66]. In our study, we found differential expression of thyroid hormone-regulated pathways and identified upregulation of DIO3 in TG2^−/−^ beige cells, which inactivates T3. We found that the free bioactive fT3 concentration was significantly lower in the differentiation media of TG2^−/−^ beige cells as compared with the TG2^+/+^ controls, in line with the higher expression of DIO3 in this cell type. T3 plays a crucial role in the activation of the browning process and differentiation of beige adipocytes [67,68,69], and it is a standard component of the differentiation medium. It is important to note that we did not add T4 to the culture media that the cells could utilize. The association of higher DIO3 expression with lower fT3 concentration raises the possibility that this enzyme may contribute to the lower mitochondrial functions of the TG2^−/−^ beige genotype.

We also identified three downregulated mitochondrial solute transporters. The most important is the SLC25A45 transporting acylcarnitine, ATP, and amino acids that cross the inner mitochondrial membrane into the mitochondrial matrix for further processes [52,53,54]. The expression of SlC25A45 was significantly lower both in TG2^−/−^ preadipocytes and beige cells which probably has an essential role in the development of the hypometabolic phenotype of these cells. Importantly, the expression of SLC25A45 can be activated by T3 as a putative thyroid response element was found in the promoter region of its gene [70], that is, DIO3-mediated T3 inactivation can contribute to its observed downregulation. Interestingly, the expression of this protein was lower in the whole EWAT tissue of TG2^−/−^ animals as well. An important paralog of SLC25A45 is SLC25A47, which was also found downregulated in TG2^−/−^ beige adipocytes. Furthermore, we detected the lower expression of SLC25A42 which is responsible for CoA and ADP transport into the mitochondria [41,42,43]. The importance of our results is further enhanced by the fact that SLC25A42 has been described as a browning marker in mice and probably has a function in making fatty acids available as acyl-CoA molecules for beta-oxidation, which is the most intertwined process in the heat generation of beige cells [71]. In addition, a lower level of ADP in the matrix was significantly reflected by decreased levels of ATP in the TG2^−/−^ cells we presented.

## 4. Materials and Methods

### 4.1. Materials

All chemicals were from Sigma-Aldrich (Munich, Germany) except indicated otherwise.

### 4.2. Mice, Treatments, and Obtained Samples

TG2^−/−^ mice [18] and TG2^+/+^ littermates with C57BL/6J genetic background were obtained from heterozygous breeding couples and were genotyped in the Animal Core Facility at the University of Debrecen. Mice were housed separately, had ad libitum access to water and chow, and were kept in a 12 h dark/light cycle at 22 ± 1 °C. The 16-week-old males were anesthetized, and the stromal vascular fractions (SVFs) were isolated from the EWAT of TG2^+/+^ and TG2^−/−^ mice. SVFs containing preadipocytes were cultured in advanced Dulbecco’s (DMEM)/F12 medium supplemented with 10% fetal bovine serum (FBS), 100 U/ml penicillin-streptomycin, 33 μM biotin, and 17 μM pantothenic acid. Beige adipocyte differentiation was induced and performed as described by Kajimura’s Lab in the Supplementary Materials of their paper [31]. The average size of lipid droplets and the total lipid content of beige cells were analyzed using the Image J open-source software (version 1.51k, National Institutes of Health, Bethesda, MD, USA) and presented as area values identified on 3-3 microscopic images taken from samples of 3 TG2^+/+^ and 3 TG2^−/−^ animals. All the animal experiments were carried out according to national, and EU ethical guidelines (license numbers 14/2010/DEMAB and 1/2014/DEMAB).

### 4.3. Gene Expression Studies

Total RNA was isolated from preadipocytes and beige cells using TRIzol Reagent (Invitrogen Corporation, Carlsbad, CA, USA), according to the manufacturer’s instructions, and nucleic acid concentration was quantified by spectrometry. A High Capacity cDNA Reverse Transcription Kit (Applied Biosystems, Foster City, CA, USA) was used to generate cDNA from isolated RNA fractions. The mRNA expression levels were determined by RT-qPCR in a LightCycler 480 (Roche Diagnostics, Mannheim, Germany). In the cases of genes *Pref1*, *Ucp1*, *Tbx1*, *Tnfrsf9*, and *Tmem26*, Maxima SYBR Green/ROX qPCR Master Mix (Thermo Fisher Scientific, Waltham, MA, USA) was used by applying a program of 10 min at 95 °C, followed by 50 cycles of 10 s at 95 °C, 1 min at 60 °C, and 50 s at 72 °C. Single-product amplification was verified by an integrated post-run melting curve analysis. Primers are listed in the Supplementary materials (Supplementary Table S1). Gene expression levels of *Slc25a45*, *Slc25a42*, *Slc25a47*, *Dio3*, *Bnip3*, *Cxcl1*, and *Trpv1* were determined using qRT-PCR applying FAM-MGB labeled Taq-Man probes (Thermo Fisher Scientific, Waltham, MA, USA) on Roche Light Cycler 480 platform (Roche Diagnostics, Mannheim, Germany). Taqman™ assays are listed in Supplementary materials (Supplementary Table S2). Gene expression was quantified by the comparative C_p_ method and normalized to the cyclophilin housekeeping gene. The values are expressed as mean ± SD of the mean calculated from 3 technical replicates for each sample of 3 TG2^+/+^ and 3 TG2^−/−^ animals.

### 4.4. Western Blots

Frozen samples were heat-treated in 2× Laemmli buffer (5 min, 100 °C) and sonicated using a Branson Sonifier 450 (Emerson Electric Co., St. Louis, MO, USA) for 2 min at maximum intensity, and cycle control of 40%. It was followed again by heat treatment (5 min, 100 °C) and (15 min, 14,000× *g*). Proteins were separated on 10% or 12% SDS-PAGE, blotted onto a PVDF membrane, and blocked using 4% non-fat dried milk in PBS. The membranes were probed by primary antibodies overnight at 4 °C, followed by incubation with horseradish-peroxidase (HRP)-conjugated species-corresponding secondary antibodies for 1 h at room temperature. Immunoblots were developed with Immobilon Western chemiluminescent substrate (Advansta, San Jose, CA, USA). Densitometry was carried out using the Image J open-source software (version 1.51k, National Institutes of Health, Bethesda, MD, USA), and the expression of proteins was normalized to actin expression (relative optical density, ROD). The source and dilution of the primary and secondary antibodies are listed in Table 1. The experiments were performed using samples of 3 TG2^+/+^ and 3 TG2^−/−^ animals, and the expression values are presented as mean ± SD of the mean.

### 4.5. Determination of Mitochondrial Dehydrogenase Activity

3-(4,5-Dimethylthiazol-2-yl)-2,5-diphenyl tetrazolium bromide (MTT) was added to the confluent cells grown in 12-well plates in 0.5 mg/mL concentration and incubated for 120 min at 37 °C. Supernatants were aspirated and replaced by DMSO (50 μL/well). Measurements were performed using a Synergy Multimode Microplate Reader (BioTek Instruments, Inc., Winooski, VT, USA) at 540 nm, as described in [72]. The values are expressed as mean ± SD of the mean calculated from samples of 3 TG2^+/+^ and 3 TG2^−/−^ animals.

### 4.6. In Vitro Cell Proliferation Assays

Cellular proliferation was determined using a sulforhodamine B (SRB) assay, as described in [73]. Cells were plated in 8-well plates and fixed in 10% trichloroacetic acid (TCA), then incubated for 1 h at 4 °C. Plates were washed 5 times with distilled water and stained with 0.4% (*w*/*v*) SRB solution in 1% acetic acid. Unbound dye was removed by washing 5 times with 1% acetic acid. The protein-bound stain was solubilized in 10 mM Tris base, and the absorbance was measured using a Synergy Multimode Microplate Reader (BioTek Instruments, Inc., Winooski, VT, USA) at 515 nm. The values are expressed as mean ± SD of the mean calculated from samples of 3 TG2^+/+^ and 3 TG2^−/−^ animals.

### 4.7. Measurement of Oxygen Consumption and Extracellular Acidification Rate

Oxygen consumption rate (OCR) and extracellular acidification rate (ECAR) were measured using an XF96 analyzer (Seahorse Biosciences, North Billerica, MA, USA), according to the manufacturer’s instructions. Cells were seeded in 96-well Seahorse assay plates. First, we detected baseline OCR and calculated ECAR. Then, proton leak respiration, which reflects heat production, was measured by adding 2 µM oligomycin, which blocks the ATP synthase. As the last step, for baseline correction, cells received a single bolus of 10 µM antimycin A treatment. OCR and ECAR were recorded every 30 min. The data were normalized to protein content measured using a BCA Protein Assay Kit (Thermo Fisher Scientific, Rockford, IL, USA). The values are expressed as mean ± SD of the mean calculated from samples of 4 TG2^+/+^ and 4 TG2^−/−^ animals.

### 4.8. Measurement of NADH and ATP Levels

Relative NADH content was determined using an NADH Quantitation Kit (Sigma-Aldrich, Munich, Germany), according to the manufacturer’s instructions. Relative ATP content was determined with an ATP Bioluminescence Assay Kit II (Roche Diagnostics, Mannheim, Germany), according to the manufacturer’s instructions. The NADH and ATP levels were normalized to protein content measured using a BCA Protein Assay Kit (Thermo Fisher Scientific, Rockford, IL, USA). The values are expressed as mean ± SD of the mean calculated from samples of 3 TG2^+/+^ and 3 TG2^−/−^ animals.

### 4.9. Immunocytochemistry, Image Acquisition, and Analysis

Cells were plated in 96-well plates. As a control, we treated the cells with the Complex III inhibitor antimycin A (10 µM). Cells were stained with MitoTracker Deep Red (Thermo Scientific, MA, USA) in 300 nM final concentration. The cells were washed in PBS and fixed in 4% paraformaldehyde for 15 min, and then permeabilized using 1% Triton X-100 in PBS for 10 min at room temperature. The cells were blocked with 1% bovine serum albumin (BSA) in PBS for 1 h and incubated with TexasRed-X Phalloidin (T7471, 1:150, Thermore Fisher, Waltham, MA, USA) for 1 h at 4 °C. Cells’ nuclei were visualized with DAPI (R37605, 1:10, Thermo Fischer Scientific Inc., Rockford, IL, USA) and rinsed in PBS twice for 10 min.

Images were made on an Opera Phoenix High Content Screening System (Perkin-Elmer, Waltham, MA, USA). Images were taken from all three channels in 25 fields per well, resulting in a total of 2400 fields covered in the 96-well plates with a non-confocal fluorescent microscope. Then, the raw images were loaded into Ilastik (European Molecular Biology Laboratory, Heidelberg, Germany) for pixel classification [74] and to segment the nuclei from the DAPI channel, the cytoplasms, and out-of-focus areas from the TexasRed Phalloidin channel. Cells were eligible for analysis only if they were not overlapping with an out-of-focus object. In the case of having an out-of-focus object inside the cell, all the previously segmented components of the cell, i.e., the related nucleus, cytoplasm, and mitochondrial classes, were discarded from the analysis. The resulting segmentation was exported to measure segmented objects in CellProfiler [75] (Broad Institute, Cambridge, MA, USA). The values are expressed as mean ± SD of the mean calculated from samples of 4 TG2^+/+^ and 4 TG2^−/−^ animals.

### 4.10. Detection of Reactive Oxygen Species Production

The level of total ROS production was measured using a luminol-chemiluminescence assay with L-012 dye (Wako Pure Chemical Industries, Ltd., 1-2 doshomachi 3-chome, Chuo-ku, Osaka, Japan) after the induction of ROS generation with 50 nM phorbol 12-myristate 13-acetate (PMA), in a reaction volume of 100 μL medium containing cells and 50 μM L-012 dye. After 5 min, samples were measured in a Synergy Multimode Microplate Reader (BioTek Instruments, Inc., Winooski, VT, USA). Production of light generated by the reaction was recorded in relative luminescence units (RLUs) and was normalized to the protein concentration levels measured using a BCA Protein Assay Kit (Thermo Fisher Scientific, Rockford, IL, USA) [76,77]. To measure the endogenous ROS production without any external induction, we used dichlorodihydrofluorescein diacetate (DCFH-DA) [78]; DCFH-DA is converted to the polar derivative DCFH by cellular esterases that are switched to highly fluorescent DCF when oxidized by intracellular ROS and other peroxides. Accumulation of DCFH in cells was measured by an increase in fluorescence at 530 nm when the samples were excited at 485 nm. Measurements were performed with a Synergy Multimode Microplate Reader (BioTek Instruments, Inc., Winooski, VT, USA) and the results were normalized to the protein content of the samples measured using a BCA Protein Assay Kit (Thermo Fisher Scientific, Rockford, IL, USA). The values are expressed as mean ± SD of the mean calculated from samples of 4 TG2^+/+^ and 4 TG2^−/−^ animals.

### 4.11. Laser Scanning Cytometry

Laser scanning microscopy (LSC) was used for fluorescent imaging with parallel fluorescent detection. The iCys imaging system is based on an Olympus IX-71 inverted microscope (Olympus Corporation, Tokyo, Japan) equipped with four lasers (405 nm, 488 nm, 561 nm, and 633 nm) two photodiodes for chromatic absorbance detection, and four photomultipliers for fluorescence detection. Scanning of the samples by laser beams took place point-by-point. During the scan, a fixed offset from the bottom of the coverslip was applied, which placed the focus on the middle plane of the cells. User-defined areas in the specimen with optimal cell density (the confluence was up to 90%) were marked as regions of interest and scanned in an automated manner. The ROI size was 10 × 10. The resolution was 1000 × 768, the pixel size was 2.5 × 2.5 µm, the field size was 250 × 192 µm, and the X-step size was 0.25 µm. For LSC imaging, a slide-based laser-scanning iCys Research Imaging Cytometer (Thorlabs Imaging Systems, Sterling, VA, USA) was used. Preadipocytes and differentiated beige cells were plated to 8-well Ibidi plates (Ibidi GmbH, Planegg/Martinsried, Germany) coated with collagen. We labeled the nuclei with 2 µg/ml DAPI and we also treated the cells with DAPI together in the presence of 10 µM antimycin A. Mitochondrial membrane potential was measured using MitoTracker Deep Red (Thermo Scientific, MA, USA) in 300 nM final concentration. The excitation wavelength was 405 nm and 633 nm and the arising fluorescent signals were collected by a 40× (NA 0.75) objective into 4 detection channels blue, long red, 570 Sp, and open channel, in both cases [79]. The values are expressed as mean ± SD of the mean calculated from samples of 4 TG2^+/+^ and 4 TG2^−/−^ animals.

### 4.12. RNA-Sequencing

To investigate global transcriptome data, high-throughput mRNA sequencing analysis was performed on the Illumina sequencing platform, as described in [80]. Genes related to mitochondrial functions and the browning process were selected using BATLAS (http://green-l-12.ethz.ch:3838//BATLAS/ accessed on 1 March 2022) and PROFAT (http://ido.helmholtz-muenchen.de/profat/ accessed on 1 March 2022) tools. The selected genes were analyzed using PANTHER (http://www.pantherdb.org accessed on 1 March 2022). The measurements were performed from samples of 3 TG2^+/+^ and 3 TG2^−/−^ animals.

### 4.13. Measurement of Free Triiodothyronine

The concentration of free triiodothyronine (fT3) in the cell culture supernatants was measured in triplicates using Cobas ECLIA kits (Roche Boehringer-Mannheim, Mannheim, Germany) with the Elecsys 2010 analyzer, according to the manufacturer’s recommendations [81,82]. The values are expressed as mean ± SD of the mean calculated from samples of 3 TG2^+/+^ and 3 TG2^−/−^ animals.

### 4.14. Statistical Analyses

GraphPad Prism version 8.1 and Microsoft Excel 14.0 were used for data interpretation and calculation of significance. Results are represented as average ± SD unless stated otherwise. For comparing groups, Student’s *t*-test and two-way ANOVA (Tukey’s multiple comparison test) were used. Values of *p* < 0.05 were considered to be statistically significant with *, **, and *** corresponding to *p* < 0.05, *p* < 0.01, and *p* < 0.001, respectively.

## 5. Conclusions

Through increasing energy expenditure, pharmacological activation of the browning process may contribute to the treatment of obesity and associated diseases in the future. Our data suggest that TG2 plays a critical role in controlling the thermogenic capacity of beige adipocytes. The absence of TG2 makes preadipocytes of EWAT hypometabolic and activators of beige differentiation cannot compensate for this phenomenon completely. The result is a beige cell in which the mitochondria probably cannot reach the threshold required for normal thermogenic functions which may contribute to the previously described decreased cold tolerance of TG2^−/−^ mice [25]. The presented data have added TG2-related regulatory processes to the list of possible targets for interventions in obesity and related metabolic disorders. However, further investigation is needed on how TG2 plays a role in the observed event through its possible substrates and/or interacting partners.

## Figures and Tables

**Table 1:** List of antibodies used in Western blot experiments.

Antibody	Dilution	Suppliers
β-ACTIN	1:5000	Sigma Aldrich (#A2066)
UCP1	1:1000	Sigma Aldrich (#SAB1404511)
TOMM20	1:1000	Abcam (#ab56783)
PGC1α	1:1000	Santa Cruz Biotechnology (#D1112)
Phospho-AMPKα (Thr172)	1:1000	Cell Signaling (#2535)
OXPHOS	1:1000	Abcam (#ab110411)
OPA1	1:1500	Novus Biologicals (#NB110-55290)
MFN2	1:1000	Sigma Aldrich (#WH0009927M3)
MFF	1:1000	Proteintech (#17090-1-AP)
DRP1	1:1000	BD Biosciences (#611112)
Anti-rabbit IgG, HRP conjugated secondary antibody	1:10,000	Advansta (R-05072-500)
Anti-mouse IgG, HRP conjugated secondary antibody	1:10,000	Advansta (R-05071-500)

## Data Availability

The BioProject’s metadata on RNSaseq experiments is available at https://dataview.ncbi.nlm.nih.gov/object/PRJNA774951?reviewer=7st5gcmn5pf4qgqn0o2ga47esd (accessed on 1 March 2022).

## Supplementary Materials

The following supporting information can be downloaded at: https://www.mdpi.com/1422-0067/23/9/5175/s1 [83,84].
