# Supplementary material for: Tissue Transglutaminase Knock-Out Preadipocytes and Beige Cells of Epididymal Fat Origin Possess Decreased Mitochondrial Functions Required for Thermogenesis
Source: Int J Mol Sci. 2022 May 5;23(9):5175. doi: 10.3390/ijms23095175 (PMC9105016; doi:10.3390/ijms23095175)
Supplement: Supplementary file 1 [file ijms-23-05175-s001.zip › ijms-1685777-supplementary.pdf]

Supplementary Materials

Supplementary methods

List of primers and reagents used in gene expression studies

Table S1. Primers of RT-qPCR reactions using SYBR.

Gene Symbol	Forward Primer (5'–3')	Reverse Primer (5'–3')
Pref1	CGGGAAATTCTGCGAAATAG	TGTGCAGGAGCATTCGTACT
Ucp1	GGCCTCTACGACTCAGTCCA	TAAGCCGGCTGAGATCTTGT
Tbx1	TTTGTGCCCGTAGATGACAA	CTCGGCCAGGTGTAGCAG
Tnfrsf9	CGTCTGTCGACCCTGGAC	CACGTCCTTCTCCGTGGT
Tmem26	CTGCTCAACCTCTTGCTGGT	AAGATGGCCGGAGAAAGC

Table S2. List of the Taqman™ assays used for the RT-qPCR measurements.

Gene Symbol	Assay
Slc25a45	Mm00460303_m1
Dio3	Mm00548953_s1
Slc25a42	Mm01349122_m1
Slc25a47	Mm01327900_m1
Bnip3	Mm00833810_g1
Cxcl1	Mm04207460_m1

Trpv1	Mm01246302_m1
Cyclophilin (PPIA)	Mm02342430_g1

Investigation of the ultrastructure, number, and area of mitochondria using Transmission Electron Microscopy

Cells were processed for electron microscopic investigation as described [1] with minor modifications. Preadipocytes were fixed in 3% glutaraldehyde dissolved in 0.1 M cacodylate buffer (pH 7.4) containing 5% sucrose for 6 h at room temperature. After washing in 0.1 M cacodylate buffer (pH 7.4), cells were post-fixed in 1% osmium tetroxide dissolved in 0.1 M cacodylate buffer (pH 7.4) for 1 h. Then, cells were dehydrated with a graded ethanol series. Samples were embedded into DurcupanACM resin (Sigma-Aldric, Munich, Germany). Ultrathin sections were cut with Leica Ultracut UCT Ultramicrotome (Leica Microsystems, Wetzlar, Germany), collected on Formvar-coated single-slot grids, and counterstained using uranyl acetate and Reynolds lead citrate. Sections were examined with a JEOL 1010 transmission electron microscope (JEOL Ltd., Tokyo, Japan) and photographed with an Olympus Veleta CCD camera (Olympus, Tokyo, Japan). The morphometric assessment was accomplished using the ImageJ software. EM pictures of at least 60 different cells of each group were analyzed and cytosolic electron-dense particles were counted.

Determination of amino acids in the cell culture supernatant

200 µl sample from the cell culture supernatants were transferred to a Nanosep3K (Sigma Aldrich, Munich, Germany) size-exclusion spin column to separate the amino acids from the proteins and

molecules with higher molecular weight. Samples were centrifuged with 14.000 RPM at 4ºC for 5 minutes and the flow-throughs were subjected to chemical derivatization using AccQ-tag derivatization reagent (Waters Corporation, Milford, MA, USA) according to the manufacturer's instruction. Briefly, 10 µl samples were mixed with 20 µl AccQ-tag derivatization reagent and with 70 µl borate buffer (pH 8.8) and incubated at 55ºC for 10 minutes [2]. The derivatized amino acids were subjected to UPLC-UV analysis using 1- and 10-times dilutions. Liquid chromatographic separation was performed on an Acquity H-class UPLC system (Waters Corporation, Milford, MA, USA) controlled by the Empower 3 software (Waters Corporation, Milford, MA, USA). The separation of the derivatized amino acids was carried out on an AccQ-tag Ultra C18 column (1.7 µm; 2.1x100 mm, Waters Corporation, Milford, MA, USA) guarded by an Acquity in-line filter (0.2 µm; 2.1 mm, Waters Corporation, Milford, MA, USA). The flow rate was set to 0.6ml/min and the column heater worked on 43ºC. Solvent A was 100% AccQ-tag Ultra eluent A, solvent B was 10% AccQ-tag Ultra eluent B in LC water, solvent C was LC water and solvent D was 100% AccQ-tag Ultra eluent B. Table 1 contains the elution profile of the UPLC separation. The PDA detector was set to 260nm wavelength with 10 points/sec sampling rate. 1 µl sample was injected in duplicates for the analyses. For quantification of the amino acids, a 6 point calibration curve was created in the range of 2.5-25 pmol/µl concentration using a mixture of the 20 proteinogenic amino acids. The quantification of the amino acids was carried out by the Empower 3 software.

Supplementary Figures

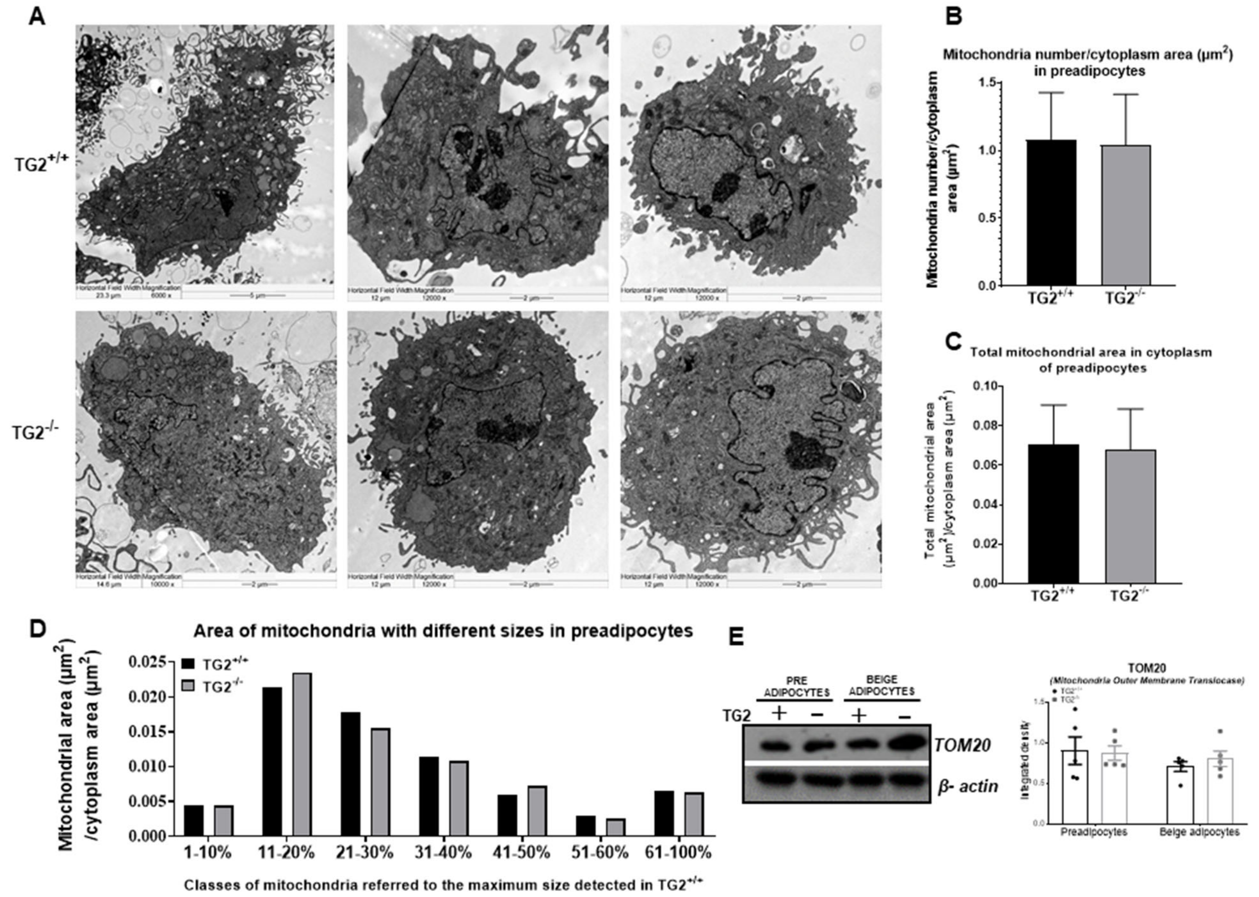

Figure S1. The morphology and size of mitochondria are similar in TG2$^{-/-}$ preadipocytes compared to TG2$^{+/+}$. **A** Representative electron microscopy images of preadipocytes (magnifications: 6000x, 10000x, 12000x). **B-C** Quantification of the number of mitochondria and the total mitochondrial area (μm^2) in the cytoplasm of preadipocytes (n=60 cells per genotype). **D** Classification of mitochondria according to the size. The largest size detected in the TG2$^{+/+}$ cells was taken as 100%. **E** Representative Western blot and quantitative analyses of TOM20. Statistical analyses were performed using Student's t-test. Data are represented as mean values ± SD.

A

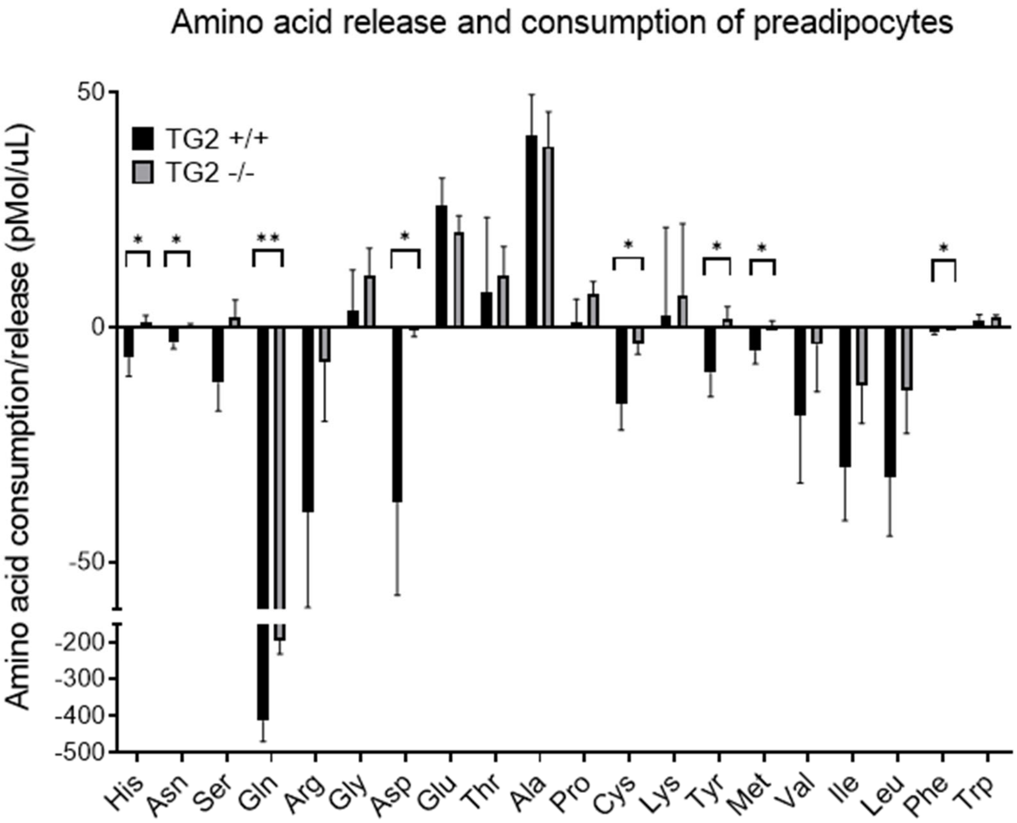
Amino acid release and consumption of preadipocytes
TG2 +/+
TG2 -/-
Amino acid consumption/release (pMol/uL)
50
0
-50
-200
-300
-400
-500
His
Asn
Ser
Gln
Arg
Gly
Asp
Glu
Thr
Ala
Pro
Cys
Lys
Tyr
Met
Val
Ile
Leu
Phe
Trp
*
**

B

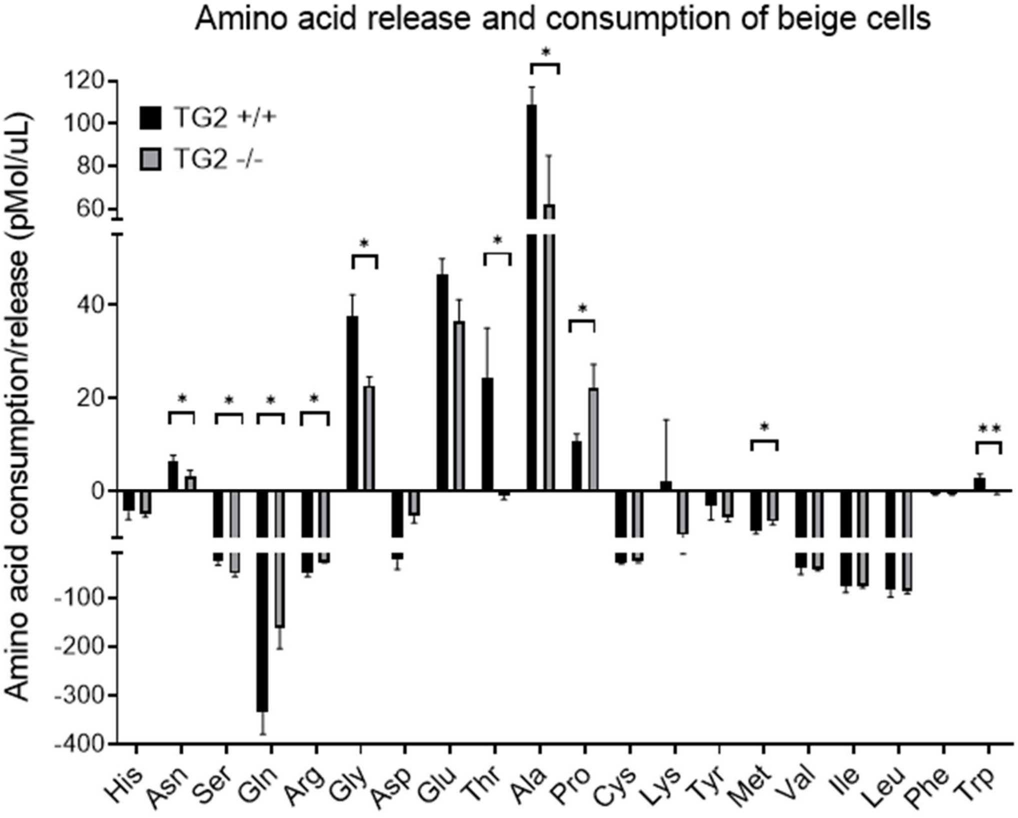
Amino acid release and consumption of beige cells
TG2 +/+
TG2 -/-
Amino acid consumption/release (pMol/uL)
120
100
80
60
40
20
0
-100
-200
-300
-400
His
Asn
Ser
Gln
Arg
Gly
Asp
Glu
Thr
Ala
Pro
Cys
Lys
Tyr
Met
Val
Ile
Leu
Phe
Trp
*
**

Figure S2: Amino acid release and consumption of TG2$^{-/-}$ and TG2$^{+/+}$ cell **A** Amino acid release (positive values) and consumption (negative values) of preadipocytes and **B** beige cells**.** Columns represent the mean values ± SD. Statistical analyses were performed using Student's t-test (n = 3, *p < 0.05 **p<0.01).

References

1. Nagy, L.; Márton, J.; Vida, A.; Kis, G.; Bokor, É.; Kun, S.; Gönczi, M.; Docsa, T.; Tóth, A.; Antal, M.; Gergely, P. Glycogen phosphorylase inhibition improves beta-cell function. *Br. J. Pharmacol*. **2018**, *175*, 301–319.
2. Armenta, J.M.; Cortes, D.F.; Pisciotta, J.M.; Shuman, J.L.; Blakeslee, K.; Rasoloson, D.; Ogunbiyi, O.; Sullivan, D.J.; Shulaev, V. A sensitive and rapid method for amino acid quantitation in malaria biological samples using AccQ•Tag UPLC-ESI-MS/MS with multiple reaction monitoring. *Anal. Chem*. **2011**, *82*, 548–558.
